# Effects of irradiance and prey deprivation on growth, cell carbon and photosynthetic activity of the freshwater kleptoplastidic dinoflagellate *Nusuttodinium* (= *Gymnodinium*) *aeruginosum* (Dinophyceae)

**DOI:** 10.1371/journal.pone.0181751

**Published:** 2017-08-01

**Authors:** Kirstine Drumm, Mette Liebst-Olsen, Niels Daugbjerg, Øjvind Moestrup, Per Juel Hansen

**Affiliations:** Marine Biological Section, Department of Biology, University of Copenhagen, Copenhagen, Denmark; University of Cambridge, UNITED KINGDOM

## Abstract

The freshwater dinoflagellate *Nusuttodinium aeruginosum* lacks permanent chloroplasts. Rather it sequesters chloroplasts as well as other cell organelles, like mitochondria and nuclei, from ingested cryptophyte prey. In the present study, growth rates, cell production and photosynthesis were measured at seven irradiances, ranging from 10 to 140 μmol photons m^-2^s^-1^, when fed the cryptophyte *Chroomonas* sp. Growth rates were positively influenced by irradiance and increased from 0.025 d^-1^ at 10 μmol photons m^-2^s^-1^ to maximum growth rates of ~0.3 d^-1^ at irradiances ≥ 40 μmol photons m^-2^s^-1^. Similarly, photosynthesis ranged from 1.84 to 36.9 pg C cell^-1^ h^-1^ at 10 and 140 μmol photons m^-2^s^-1^, respectively. The highest rates of photosynthesis in *N*. *aeruginosum* only corresponded to ~25% of its own cell carbon content and estimated biomass production. The measured rates of photosynthesis could not explain the observed growth rates at high irradiances. Cultures of *N*. *aeruginosum* subjected to prey starvation were able to survive for at least 27 days in the light. The sequestered chloroplasts maintained their photosynthetic activity during the entire period of starvation, during which the population underwent 4 cell divisions. This indicates that *N*. *aeruginosum* has some control of the chloroplasts, which may be able to replicate. In conclusion, *N*. *aeruginosum* seems to be in an early stage of chloroplast acquisition with some control of its ingested chloroplasts.

## Introduction

About half the known species of free-living dinoflagellates lack built-in chloroplasts and are referred to as heterotrophic dinoflagellates. However, during the past three decades a growing number of dinoflagellate species have been shown to exploit chloroplasts from their prey and thereby supplement their carbon needs via photosynthesis [1]. Exploitation of ingested chloroplasts can be found among several dinoflagellate genera and is organized in many different ways. Some species utilize intact algal cells as ectosymbionts (e.g. *Ornithocercus* [2, 3] and *Amphisolenia* [4]) or endosymbionts (e.g. the green *Noctiluca* [5–7]). In other species, only the chloroplasts are utilized (i.e. *Dinophysis* [8, 9]), and genes allowing the dinoflagellate to utilize the chloroplasts have been transferred to the host genome [10].

In between these two extremes, species are found that sequester prey nuclei and mitochondria together with the chloroplasts, allowing the dinoflagellates to utilize the acquired chloroplasts for days or weeks. This ability has been reported from a number of freshwater species: *Nusuttodinium acidotum*, *N*. *amphidinoides and N*. *aeruginosum* [11–13], as well as marine species: *Amphidinium wigrense*, *Amylax buxus*, *A*. *triacantha*, *Cryptoperidiniopsis* sp., *Gymnodinium gracilentum*, *Nusuttodinium latum*, *N*. *myriopyrenoides*, *N*. *poecilochroum*, *Pfiesteria piscicida* and a yet undescribed Antarctic dinoflagellate [14–22]. Our knowledge of the ecophysiology of these organisms is still quite sparse. The dependency of feeding and growth on irradiance has so far only been studied in two marine species *G*. *gracilentum* and *N*. *poecilochroum* [17, 23]. These species can grow in complete darkness if supplied with fresh prey. However, their growth rates depend on irradiance, and rates increase by a factor of 2–3 when at irradiances > 50 μmol photons m^-2^s^-1^. Ingestion rates are also light dependent and the rates also increase by a factor of 2–3. This indicates that increased-light-dependent digestion rates are the explanation for the observed elevations in growth rates at higher irradiances. Rates of photosynthesis are only available for *G*. *gracilentum* at a few irradiances [17] and suggest that photosynthetic activity decreases rapidly after prey starvation. Hardly any photosynthesis could be measured in prey-starved cultures after 48h.

We found the species *N*. *aeruginosum* to be common in small lakes in Denmark, established it into culture and studied its ecophysiology. Species identification was based on light microscopy and a sequence comparison of the nuclear-encoded LSU rDNA gene (1400 base pairs) between our strain and a Japanese strain of *N*. *aeruginosum* studied by Onuma et al. [24] (Genbank accession number LC027055). The sequence divergence was 0.5% (data not shown).

In the present work we aimed to study growth, cell production and photosynthesis of the dinoflagellate at different irradiances when fed a cryptophyte identified as *Chroomonas* sp. This work represents the first ecophysiological study of a freshwater kleptoplastidic species. Specifically, we examined (1) growth and feeding responses as a function of irradiance and (2) the role of photosynthetic activity of the retained chloroplasts during prey starvation.

## Materials and methods

### Isolation and maintenance of cultures

*Nusuttodinium aeruginosum* was isolated from a surface water sample from Horsekær, Tibirke, Denmark in July 2012. *Chroomonas* sp. (SCCAP K-1623) was obtained from Scandinavian Culture Collection of Algae & Protozoa. Cells were grown in L16 medium and were maintained in TPP 24 well test plates. The cultures were kept at an irradiance of 65 μmol photons m^-2^s^-1^, a light:dark cycle of 16:8 h and temperature of 15±1°C. Irradiance was measured using a LI-COR LI-1000 radiation sensor equipped with a spherical probe.

### Light microscopy

**Fig 1:**
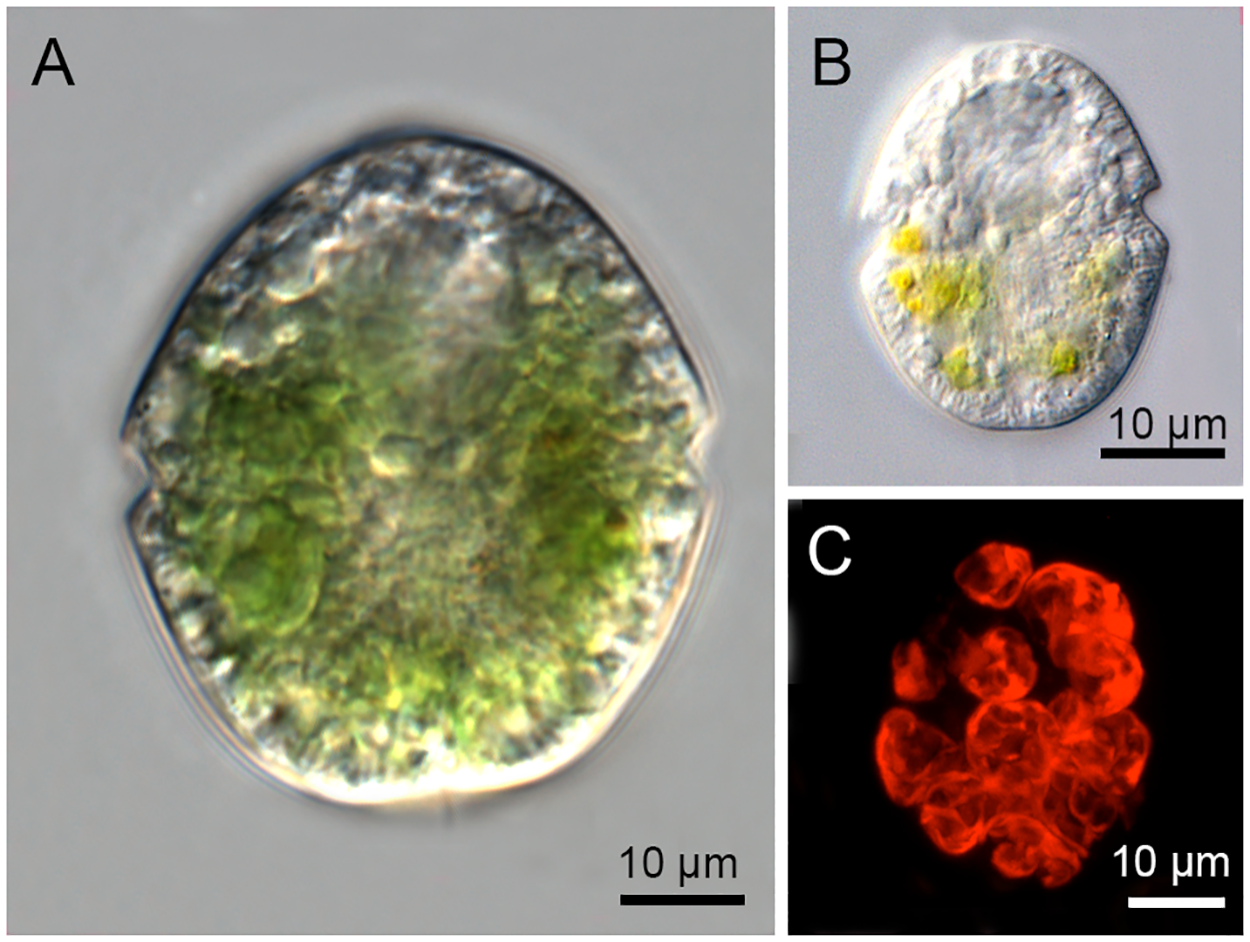
Light microscopy of *Nusuttodinium aeruginosum*. (A) Large chloroplast-containing cell. (B) Small cell showing chloroplast degradation. (C) Epifluorescence microscopy revealing numerous chloroplasts.

Live cells (Fig 1A and 1B) were observed using an Axio imager.M2 (Zeiss, Germany) equipped with Nomarski interference contrast and a x63 oil objective (NA = 1.4). Micrographs were taken with an Axiocam HRc digital camera (Zeiss, Germany).

### Epifluorescence microscopy

Well-fed cells, experiencing prey concentrations > 5000 *Chroomonas* sp. cells ml^-1^, were fixed in 2% glutaraldehyde in L16 medium and filtered onto a black filter with a pore size of 0.22 μm (Osmonic Inc). Kleptochloroplasts were examined with an Olympus IX81 motorized inverted microscope equipped with epifluorescence illumination and a disc-spinning unit for confocal imaging. Stacks of 36 images were taken of three individual cells using a digital camera (Soft Imaging System F View II). Chloroplasts from a 3D-reconstructed cell is shown in Fig 1C.

Two experiments on *N*. *aeruginosum* were conducted. The first experiment aimed to quantify the effects of irradiance on growth, photosynthesis and cell carbon while the second experiment was carried out to study the effects of prey starvation on photosynthetic activity of *N*. *aeruginosum*. Both experiments were carried out in TPP 96 well test plates.

### Experiment 1: Effects of irradiance on growth, photosynthesis and cell volume

The effect of irradiance on the growth of *Nusuttodinium aeruginosum* was studied at seven different irradiances: 10, 20, 25, 40, 65, 100 and 140 μmol photons m^-2^s^-1^. Seven plates, each containing 12 replicates of mixed cultures and 6 replicates of mono cultures of prey were initiated at the same time at the different irradiances. Each replicate was initiated with ten well-fed *N*. *aeruginosum* which were picked individually with a drawn Pasteur pipette and transferred to a well with 100 μl L16 medium. Adding 200 μl of 500 cells ml^-1^ *Chroomonas* sp.*-*culture gave an initial 10:100 relationship between predator (*N*. *aeruginosum*) and prey (*Chroomonas* sp.). Subsequently one plate was fixed for every doubling time (varied between 2 and 4 days). Cells were enumerated directly from wells using an inverted microscope. For *Chroomonas* sp. a minimum of 200 cells was counted (if the total was less than 200, then all cells were counted). All *N*. *aeruginosum* cells were counted and cell volume measured, giving datasets of 12 replicates for mixed and 6 replicates for mono cultures. Photosynthesis was measured once at each light intensity after approximately 4 generations (see below for details).

### Experiment 2: Survival and photosynthetic activity of endosymbionts during prey deprivation

The second experiment applied the same set up as experiment 1, but the initial number of *Nusuttodinium aeruginosum* was changed to 50 cells per well and prey was not added. Prior to the commencement of the experiment the predator was separated from the prey, as described under photosynthesis. To ensure that *N*. *aeruginosum* was in exponential growth phase when transferred, cells were taken on day eight from a 96-well plate which had been initialized as in experiment 1. The experiment was carried out at 65 μmol photons m^-2^s^-1^. Measurements of photosynthesis, cell numbers and cell volumes were done on day 0, 2, 4, 6, 9, 14 and 27 (see below for details).

### Measurements of growth and cell volume

The cell concentrations of *Nusuttodinium aeruginosum* were determined by total cell count of cells growing in 96-well plates. The growth rate (μ, d^-1^) was calculated using:

$$\mu_{y}=(ln_{e}\left(N_{t}-\;ln_{e}\left(N_{0}\right)\right)t^{-1}$$ (1)

where *N*_*t*_ is the number of cells at time *t* and *t* is the experimental time (days). Growth rate (*μ*_y_) as a function of irradiance was fitted to Michaelis-Menten kinetics.

Cell volume (*V*_*y*_) were computed using the geometric formula for a prolate spheroid (length > width = depth) on Lugol-fixed cells. Calculations of cell carbon (C_y_) of *N*. *aeruginosum* were based on Menden-Deuer and Lessard (2000):

$$C_{y}=0.76\times V_{y}^{0.819}$$ (2)

where *V*_*y*_ is cell volume of *N*. *aeruginosum*. Biomass production rate was computed as:

$$Biomass\;production=\mu_{y}\;\times \;C_{y}$$ (3)

where *μ*_*y*_ is growth rate (μ, d^-1^) and *C*_*y*_ is (pg C cell^-1^). As with growth rate, both cell volume, cell carbon and biomass production as functions of irradiance were fitted to Michaelis-Menten kinetics.

### Photosynthesis

Photosynthetic activity was measured by a modification of the 'single-cell method' [24, 25]. The prey *Chroomonas* sp. is photosynthetic, and it was therefore necessary to separate predators from prey prior to photosynthesis measurements. This was done by picking individual *Nusuttodinium aeruginosum* cells with a hand drawn Pasteur pipette and rinsing each cell in L16 medium. Four replicates of 23-ml glass scintillation vials were filled with 2 ml L16 medium, and 50 rinsed cells were transferred to each vial. 20 μl NaH^14^CO_3_^-^ stock solution (specific activity 100 μCi ml^-1^) was added in each vial, resulting in a specific activity of ~1.0 μCi ml^-1^. The vials were left for 3 h at the matching light intensity. Vials were accompanied by dark vials, which were treated similarly, but wrapped in tin foil during incubation. After incubation the specific activity was determined by transferring 100 μl from each vial to new vials containing 200 μl phenylethylamine. The remaining sample was acidified with 2 ml 10% glacial acetic acid in methanol and left overnight for evaporation on a 65°C heat plate. A volume of 1.5 ml distilled water was added to the dried samples, followed by 10 ml of Packard Insta-Gel scintillation cocktail, and radioactivity was determined using a Packard 1500 Tri-Carb liquid scintillation analyzer with automatic quench correction. Rates of photosynthetic activity (PA) were calculated as follows:

PA (μgC × ml−1 × h−1)=DPM × [DIC]C14a × h (4)

Where DPM is disintegrations min^-1^ml^-1^, DIC is the concentration of inorganic carbon (μg C × ml^-1^), ^14^C_a_ is the specific activity in disintegrations min^-1^ ml^-1^ and h is the incubation time in hours. To obtain daily photosynthesis, the rates per hour were multiplied by 16. DIC concentrations were measured on 1 ml subsamples using an infrared gas analyzer (ADC 225 Mk3 Gas analyzer, Analytic Development Co. Ltd., Hoddesdon, England) as described in detail elsewhere [26]. Glass vials with screw caps were used for DIC samples allowing no headspace, and the samples were analyzed within a few hours.

## Results

### Qualitative observations of *Nusuttodinium aeruginosum* fed *Chroomonas sp*. using light and epifluorescence microscopy

Well-fed cells of *N*. *aeruginosum* were 27–57 μm long and 22–46 μm wide (Fig 1A and 1B) and contained 6–10 chloroplasts (Fig 1C). The cell size and coloration decreased with starvation time, and chloroplasts in a stage of apparent degradation were observed (Fig 1B).

### Experiment 1. Effects of irradiance on growth, photosynthesis and cell volume

**Fig 2:**
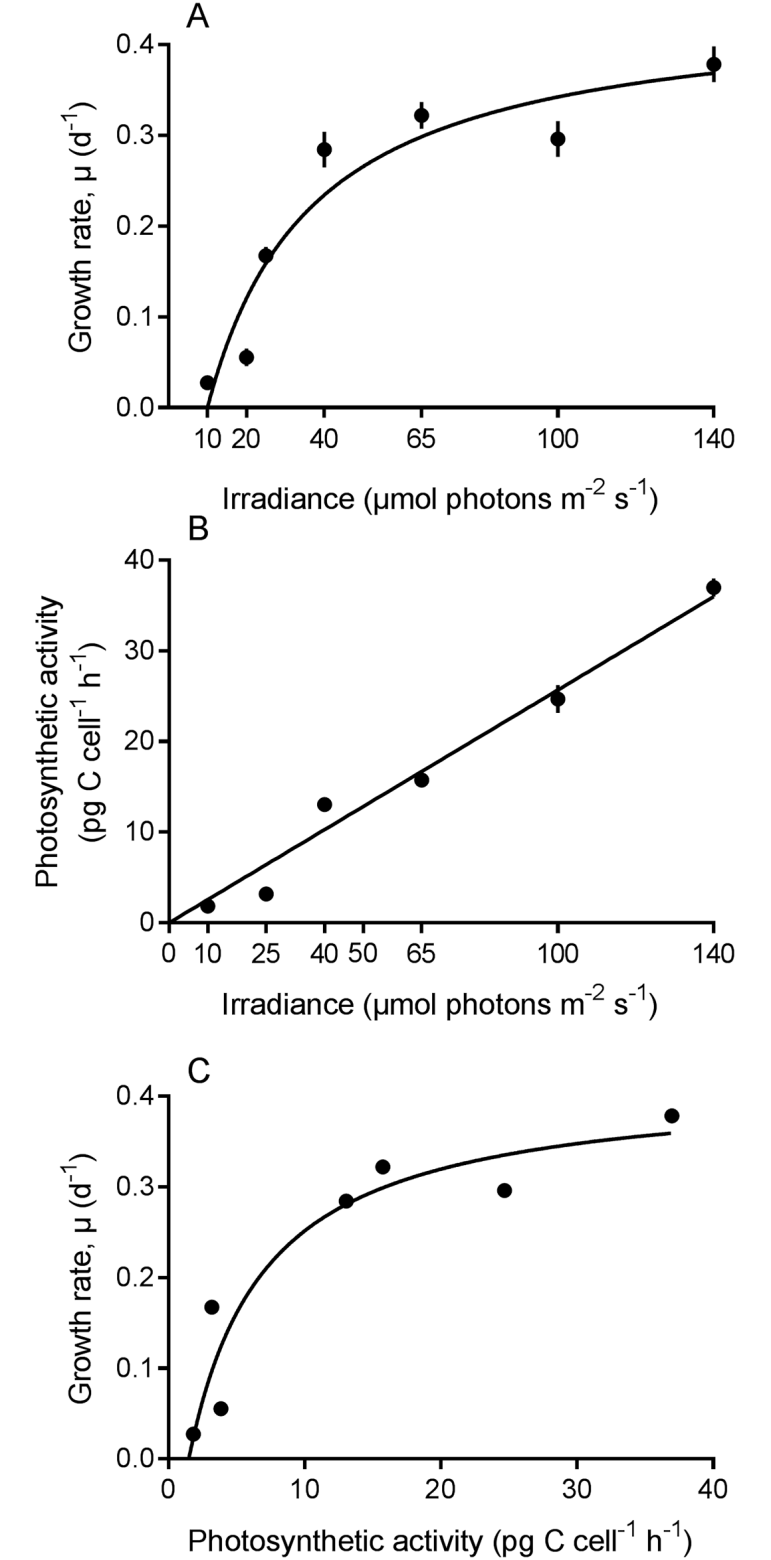
Effect of irradiance on growth and photosynthetic activity of *Nusuttodinium aeruginosum*. (A) Growth rates as a function of irradiance. The curve was numerically fitted to Michaelis-Menten kinetics. μ = 0.45*(I-10)/(26.99+(I-10)), R^2^ = 0.91. Data points represent means ± SE (n = 12). (B) Photosynthetic activity as a function of irradiance. The data was fitted to a linear line, R^2^ = 0.96. Data points represent means ± SE (n = 4, except irradiance = 10 where n = 3). (C) Growth rates of *N*. *aeruginosum* as a function of photosynthetic activity. The curve was numerically fitted to Michaelis-Menten kinetics. μ = 0.41*(I-1.5)/(5.54+(I-1.5)), R^2^ = 0.89.

The growth response of *Nusuttodinium aeruginosum* was greatly stimulated by irradiance (S1 Fig). At an irradiance of 10 μmol photons m^-2^s^-1^, the cultures more or less sustained themselves during the duration of the experiment (14 days). At higher irradiances, a short lag phase was observed, before cultures went into exponential growth, which lasted for 6–16 days (non-shaded areas in S1 Fig) depending upon irradiance. The growth rate data fitted the Michaelis-Menten kinetics closely (R^2^ = 0.91) and indicate that the growth rate of *N*. *aeruginosum* saturated at an irradiance of ~40 μmol photons m^-2^s^-1^ at a growth rate (μ) of 0.3 d^-1^ (Fig 2A).

Cell concentrations of the prey (*Chroomonas* sp.) were monitored in both mixed and mono cultures to evaluate the grazing responses of *N*. *aeruginosum* at the different irradiances. In all treatments, no significant differences were observed in prey concentrations, indicating low grazing rates (S2 Fig). To evaluate the extent to which growth rates of *N*. *aeruginosum* were affected by prey concentration at the different irradiances, the relationship between cell concentrations of *Chroomonas* sp. and growth rates of *N*. *aeruginosum* were tested (data not shown). The prey concentration increased from approximately 0.5–165.0x10^3^ cells ml^-1^, equivalent to a predator:prey ratio of 1:9 to 1:87. Only in one case, 20 μmol photons m^-2^s^-1^, a significant relationship was observed between growth rate of *N*. *aeruginosum* and concentration of the prey (p = 0.016, R^2^ = 0.95). At all other irradiances tested, the slope of the correlation was not statistically different from zero. Hence within the prey concentrations used no influence of prey concentration on *N*. *aeruginosum* (0.13 < p < 0.83) was recorded.

Photosynthetic activity of *N*. *aeruginosum* increased linearly with irradiance up to 140 μmol photons m^-2^s^-1^, which was the highest irradiance used in this experiment (Fig 2B p < 0.005, R^2^ = 0.96). The relationship between photosynthesis and growth could also be fitted to Michaelis-Menten kinetics well (R^2^ = 0.89). Thus, growth rates of *N*. *aeruginosum* saturated at a photosynthetic activity of ~12 pg C cell^-1^h^-1^ or 192 pg C d^-1^ taking the light:dark period into account (Fig 2C).

**Fig 3:**
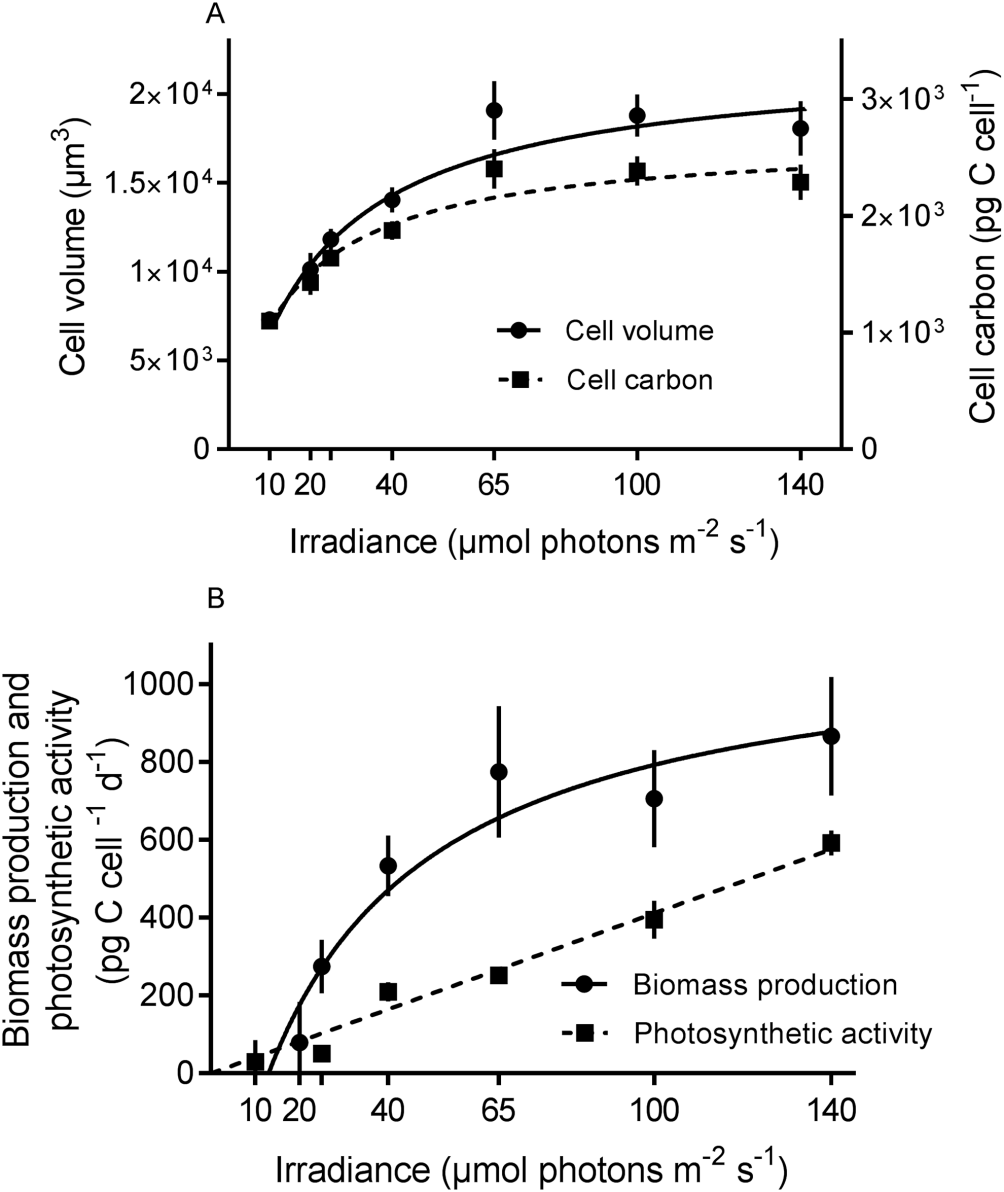
Effect of irradiance on cell volume and biomass production of *Nusuttodinium aeruginosum*. (A) Cell volume and cell carbon of *N*. *aeruginosum* as a function of irradiance. Data points represent means ± SE (n = 30–40). The curves were numerically fitted to Michaelis-Menten kinetics: cell volume = 22048*I/(21.04+I), R^2^ = 0.25; and cell carbon = 2668*I/(15.45+I), R^2^ = 0.28. Note different ordinate scales. (B) Biomass production (BP) and photosynthetic activity (PA) as a function of irradiances. The BP curve was numerically fitted to Michaelis-Menten kinetics: BP = 1144*(I-13)/(38.62+(I-13)), R^2^ = 0.93. The data for PA were fitted to a linear line, R^2^ = 0.97. Data for biomass production are the same as in Figs 2A and 3A.

Cell carbon content estimates of *N*. *aeruginosum* gave values ranging from 1.1x10^3^ ± 58 to 2.4x10^3^ ± 168 pg C cell^-1^ at 10 and 65 μmol photons m^-2^s^-1^, respectively (Fig 3A). Cell carbon and daily biomass production (growth rate x cell carbon) (Fig 3B) could be fitted to Michaelis-Menten kinetics. The low R^2^, in the cell carbon relationship is due to large variations in cell volume among cells.

### Experiment 2. Survival and maintenance of endosymbiont during prey deprivation

**Fig 4:**
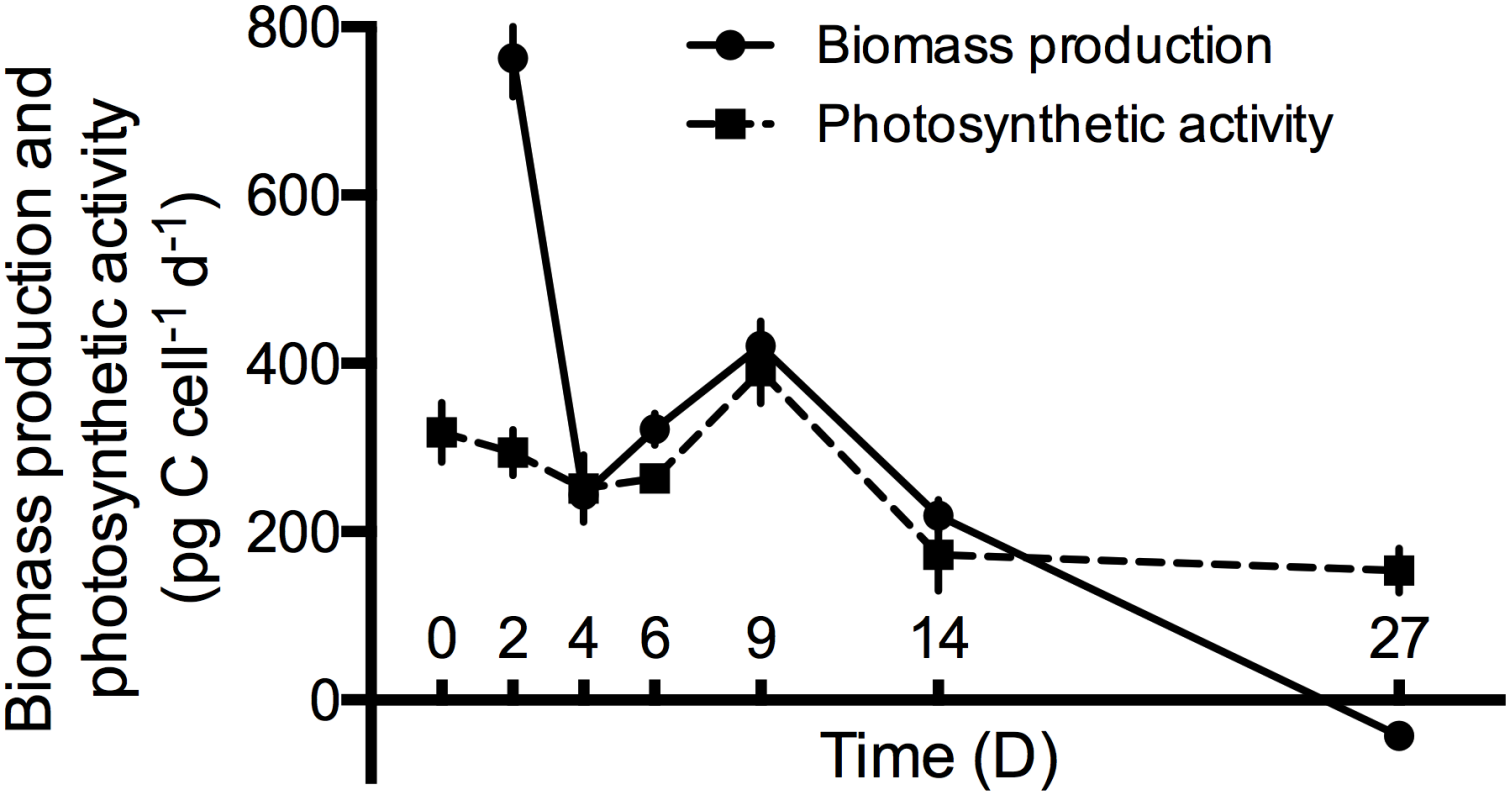
Biomass production (BP) and photosynthetic activity (PA) of *Nusuttodinium aeruginosum* as a function of time under prey deprivation. Data points represent means ± SE (BP; n = 30, PA; n = 4).

Cells of *Nusuttodinium aeruginosum* subjected to prey starvation at an irradiance of 65 μmol photons m^-2^s^-1^ divided approximately four times during the first 14 days of the experiment (S3A Fig). Net mortality of cells was observed after day 14. The cell volume of *N*. *aeruginosum* and the calculated cell carbon decreased immediately after the first cell division. From day 4 the cells remained at almost half their initial cell size in linear dimensions, which is equivalent to 1500 pg C cell^-1^ (S3B Fig). Nonetheless, there was no major decrease in the photosynthetic activity which sustained at 300 pg C cell^-1^ d^-1^ the first 10 days and stayed constant at ~160 pg C cell^-1^ d^-1^ for the remainder of the experiment (Fig 4). Biomass production was reduced (due to the close relation to cell carbon/volume) to more than half of the initial production from day 2 to 4. From then on the production showed a similar response as the photosynthetic activity. However when growth became negative so did biomass production (Fig 4).

## Discussion

### Effects of irradiance on growth and prey ingestion

Growth rates of the blue-green freshwater dinoflagellate *Nusuttodinium aeruginosum* depended strongly on irradiance. The light compensation point, where the dinoflagellate was able to sustain itself, was achieved at an irradiance of ~10 μmol photons m^-2^s^-1^. Maximum growth rates of 0.3 (d^-1^) were obtained at irradiances ≥ 40 μmol photons m^-2^s^-1^ at predator:prey ratios of 1:9 to 1:87. Data on growth rates could be fitted to Michaelis-Menten kinetics taking this light compensation point into account. Thus, *N*. *aeruginosum* is highly dependent upon light for growth and it has a growth response very similar to an entirely phototrophic species. This is unlike other closely related species, like the marine *N*. *poecilochroum* and *Gymnodinium gracilentum*, which can grow in the dark at a rate corresponding to ~40% of the maximum growth rate obtained in light [23]. While the planktonic species *N*. *aeruginosum* and *G*. *gracilentum* attain their maximum growth rates at about 50–80 μmol photons m^-2^s^-1^, the benthic species *N*. *poecilocroum* obtains its maximum growth rate at an irradiance as low as 10 μmol photons m^-2^s^-1^ [23]. The differences in responses towards light among these closely related dinoflagellates seem to reflect the light dependence of the algal prey. The prey used in the experiments on *N*. *poecilochroum* was a *Chroomonas* sp., which was well adapted to a low-light environment and sustained 50% of its maximum growth rate at only 2.9 μmol photons m^-1^s^-1^ [23].

In the present study we could not get reliable estimates of ingestion rates. The concentrations in mixed cultures were almost constantly lower than the concentration of mono cultures, but the differences between *Chroomonas* sp. in mixed and mono cultures were too small to allow for computation of ingestion rates. Since we initiated the experiments at a predator:prey ratio of 10:1, this indicates low ingestion rates (initially less than ~1–2 prey cells ingested per day).

### Importance of inorganic carbon uptake for the carbon budget

We observed a positive linear relationship between photosynthesis and irradiance within the studied irradiance levels, which is very different from the Michaelis-Menten relationship observed in the light response of growth and estimated biomass production. The potential contribution of photosynthesis varied with irradiance, with the lowest contribution at low irradiance and the highest at high irradiance. The daily inorganic carbon fixation corresponded at most to ~26% of the cell carbon content (Table 1). Reported values of inorganic carbon uptake are sparse for other dinoflagellates with kleptochloroplasts. *Dinophysis acuminata*, which only sequesters chloroplasts, has been found to have a daily specific inorganic carbon fixation corresponding to ~59% of its cell carbon content at an irradiance of 100 μmol photons m^-2^s^-1^ [27]. For dinoflagellates that also retain prey nuclei and prey mitochondria, such as the closely related species, *Gymnodinium gracilentum*, the specific inorganic carbon uptake rates were higher, ~118% [17]. Similarly high inorganic carbon fixation values (~184%) were found in the ciliate *Mesodinium rubrum*, which contains prey nuclei and mitochondria [28–30].

**Table 1:** Comparisons of maximum photosynthetic rate, cellular carbon and cell carbon fixed per day (%) in three dinoflagellate and one ciliate that all carry out acquired phototrophy. Data from the literature. All data are from experiments carried out at 15°C, except for *M*. *rubrum* (3°C).

	Irradiance (μmol photons m^-2^s^-1)^	Light:Dark cycle	P_max_^a^ (pgC cell^-1^ d^-1^)	Cellular carbon^c^ (pgC cell^-1^)	Cell carbon fixed per day (%)
*Gymnodinium gracilentum* [17]	90	16:8	123±5	104	118
*Dinophysis acuminata* [27]	100	14:10	532±84 ^b^	895±66	59
*Nusuttodinium aeruginosum* (Present study)	140	16:8	592±16	22.9x10^3^±153	26
*Mesodinium rubrum* [28]	25	16:8	726±64	597	122

^a^ P_max_ is the highest photosynthetic rate measured, it might saturate at another irradiance.

^b^ Given numbers are from well-fed cultures.

^c^ Calculated using [31]

The fact that the measured photosynthesis only to a minor degree could explain the observed increased growth rates at higher irradiances raises some questions concerning our measurements and calculations. First, cell carbon was not measured but estimated from published relationships between cell volume and cell carbon. Second, it is possible that we may have underestimated the uptake of inorganic carbon using the ^14^C technique. This may happen if the CO_2_ produced during the prey digestion is reused by the dinoflagellate. Third, it is possible that light may have a positive effect of light on growth rates. This has been documented in a number of both heterotrophic and non-constitutive mixotrophic species of ciliates and dinoflagellates [17, 32–35]. A hypothesis for this phenomenon could be that high irradiance induces and aids digestion of phytoplankton. Some studies have shown that especially phototrophic prey is more easily digested in the light [33, 36]. This is supported by a recent study which showed that the oxidation of carbohydrates by enzymes substantially increased in light [37]. Another pathway to utilize solar energy could be through the protein, rhodopsin, which was recently found in *Oxyrrhis marina* [35]. We suggest three explanations for the positive effect of light: (1) Algal pigments are highly labile in the presence of light and oxygen, making ingested material more readily utilizable for growth [38]. (2) Complex systems like enzymes or other proteins are expressed to greater extent in light. (3) The ATP produced by photosynthetic activity aids digestion and ingestion of prey, rather than to produce glucose. With increasing irradiance the photosynthesis increases, resulting in greater amounts of ATP being produced, allowing faster digestion, hence greater ingestion rates.

### *Nusuttodinium aeruginosum* during prey starvation

Previous studies have demonstrated that the kleptochloroplasts of *N*. *aeruginosum* can be retained for > 1 month, when cells are subjected to prey starvation [39]. In the present experiments, *N*. *aeruginosum* cells doubled more than 3 times during the first 9 days when subjected to prey starvation, despite this, *N*. *aeruginosum* cells were able to obtain its maximum cellular photosynthetic rate in the same period. This may indicate that *N*. *aeruginosum* is able to divide the kleptochloroplasts. However, since we did not perform either measurements of chlorophyll *a* or enumerated the chloroplasts, this is speculation and thus future experiments are needed.

Cultures of *N*. *aeruginosum* survived for more than 27 days without prey and the chloroplasts were photosynthetic active. This is a long period compared to most other dinoflagellates with kleptochloroplasts. In *Gymnodinium gracilentum* the photosynthetic activity decreased quickly with starvation time and was essentially zero after 48 h of prey starvation [17]. Similarly, although no actual rates of photosynthesis have been reported on *Pfiesteria piscicida*, autoradiographical studies have indicated photosynthetic activity in this species for a least 7 days [18]. The only dinoflagellates retaining kleptochloroplasts for longer time are species of *Dinophysis*, but they only sequester the chloroplasts, not other cell constituents [1, 27]. The ability of *N*. *aeruginosum* to retain kleptochloroplasts and other cell organelles in many ways resemble the case of the red-pigmented ciliate *Mesodinium rubrum*. This ciliate may retain and divide the kleptochloroplasts [29], using the sequestered prey nucleus to farm the chloroplasts and produce photosynthetic pigments, even performing photo acclimation [40–42].

## Conclusion

This investigation clearly shows that *Nusuttodinium aeruginosum* has some control of its kleptochloroplasts. The kleptochloroplasts were photosynthetic active for at least 27 days in prey-starved cultures, and *N*. *aeruginosum* maintained a high photosynthetic rate for the first 9 days, despite going through 3 cell divisions. These observations clearly indicate that the dinoflagellate is able to divide the sequestered chloroplasts, but details need to be investigated further. The growth rate of *N*. *aeruginosum* was strongly dependent upon irradiance, yet measured rates of photosynthesis only contributed by 15–25% of the total carbon requirements of *N*. *aeruginosum* under well-fed conditions. Thus, the sequestered chloroplasts may contribute something in addition to carbon. It is not the first time this phenomenon has been described as similar observations have been done in other heterotrophic and kleptoplastidic species [23, 33, 34]. Further studies are needed to elucidate the contribution from sequestered chloroplasts.

## Supporting information

S1 Fig Development in cell concentration of *Nusuttodinium aeruginosum* (in a mixed culture) through time under seven different irradiances (10, 20, 25, 40, 65, 100 and 140 μmol photons m^-2^ s^-1^). Data points represent 12 replicates. Shaded areas are considered lag phase and steady state, respectively and were not used in calculations of the growth rates. Arrows indicate the time of photosynthesis measurements.
(TIFF)

S2 Fig Development in cell concentration of *Chroomonas* sp. as prey (n = 12) and as control (n = 6) through time, under six different irradiances (10, 20, 25, 40, 65 and 140 μmol photons m^-2^ s^-1^). Data points represent means ± SE.
(TIFF)

S3 Fig Effect of prey deprivation on growth and cell volume of *Nusuttodinium aeruginosum*. (A) Development in cell concentration and growth rate of *N*. *aeruginosum* during prey deprivation. Data points represent means ± SE (n = 8, except day 6 and 9 = where n = 4 and 5 respectively). (B) Cell volume and cell carbon of *N*. *aeruginosum* as a function of time under prey deprivation. Data points represent means ± SE (n = 30).
(TIFF)
