# Supplementary figures and images for: Effects of irradiance and prey deprivation on growth, cell carbon and photosynthetic activity of the freshwater kleptoplastidic dinoflagellate Nusuttodinium (= Gymnodinium) aeruginosum (Dinophyceae)
Source: PLoS One. 2017 Aug 1;12(8):e0181751. doi: 10.1371/journal.pone.0181751 (PMC5538715; doi:10.1371/journal.pone.0181751)

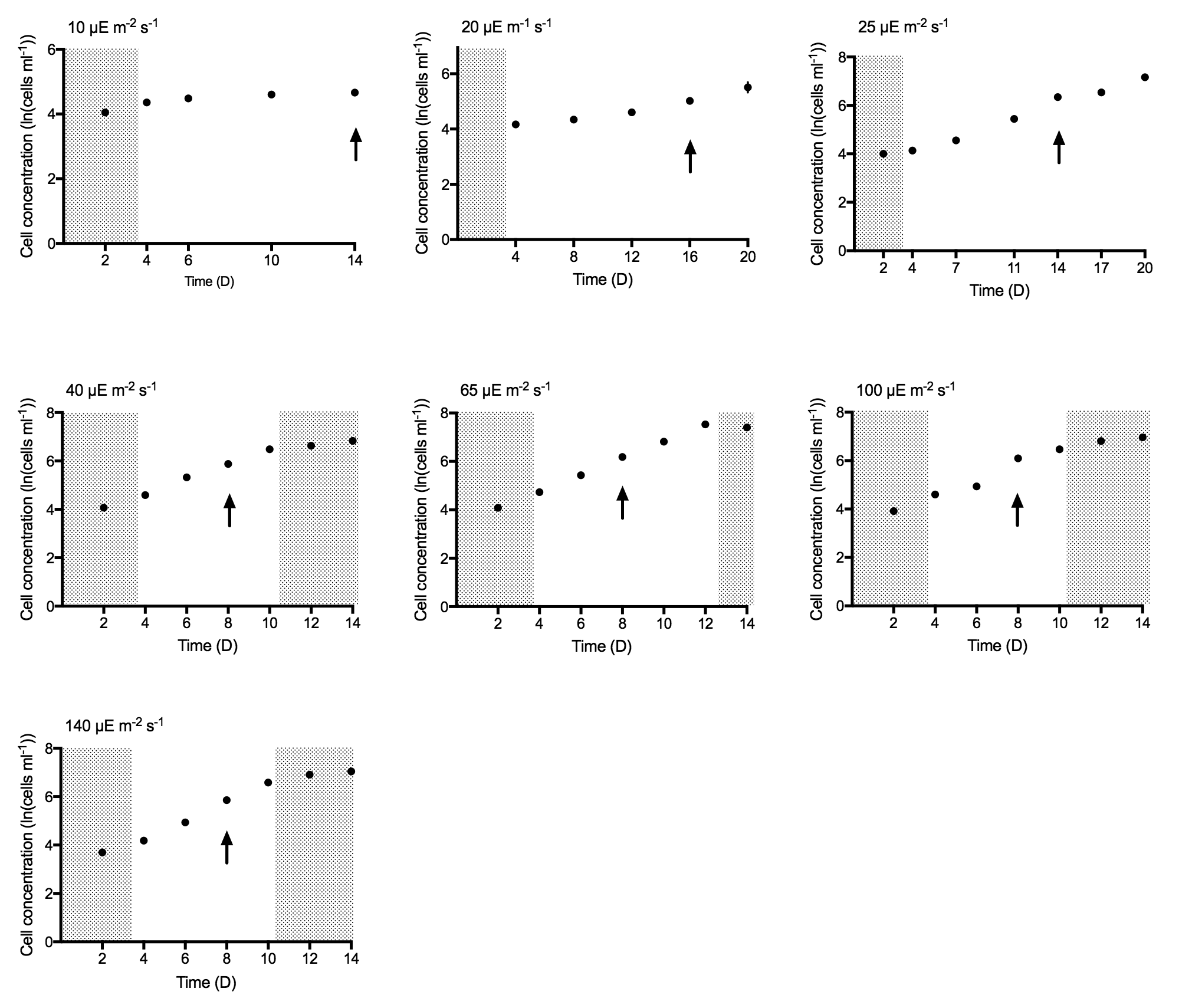

Supplement: S1 Fig — Data points represent 12 replicates. Shaded areas are considered lag phase and steady state, respectively and were not used in calculations of the growth rates. Arrows indicate the time of photosynthesis measurements. (TIFF) [file pone.0181751.s001.tiff]

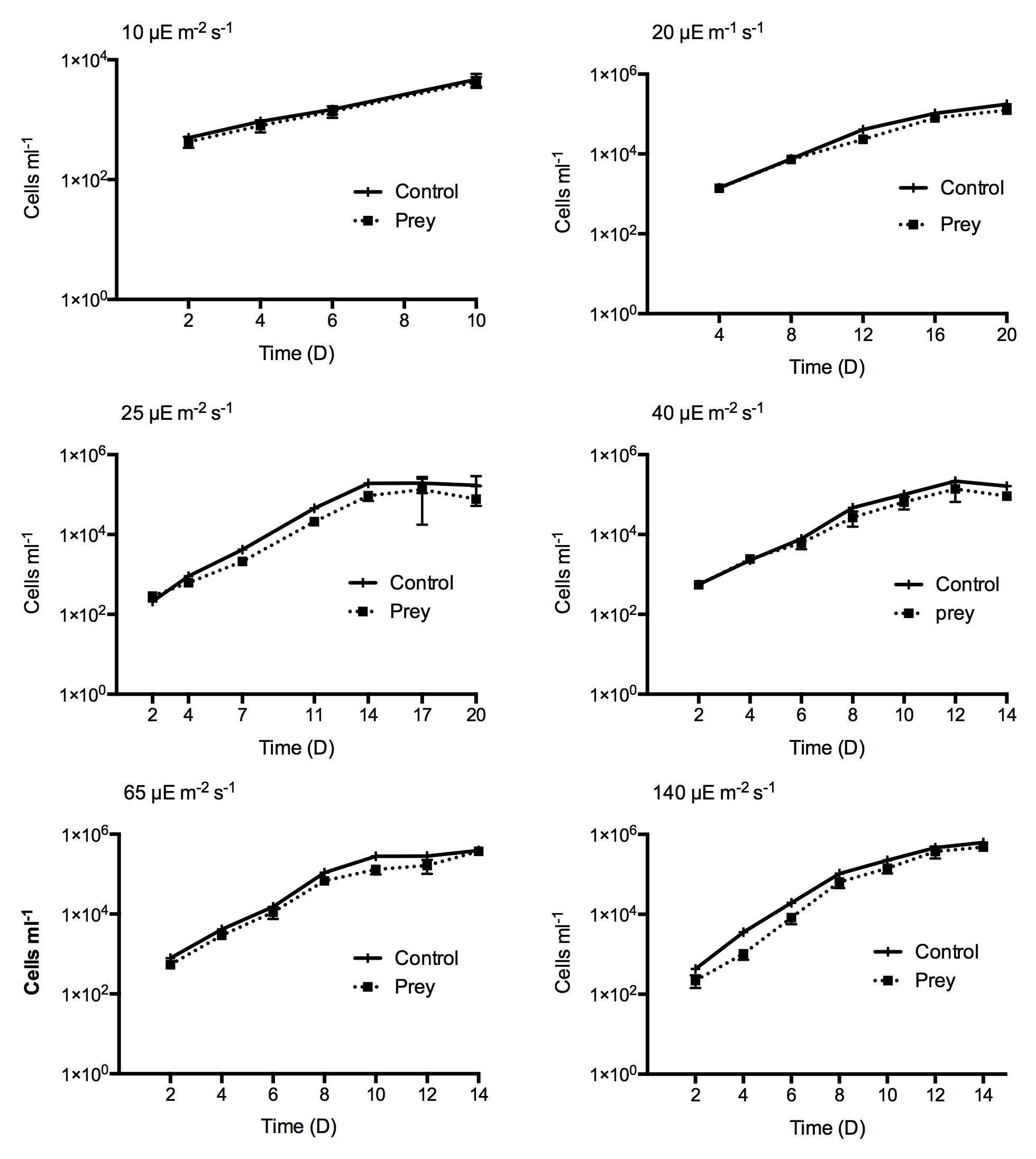

Supplement: S2 Fig — Data points represent means ± SE. (TIFF) [file pone.0181751.s002.tiff]

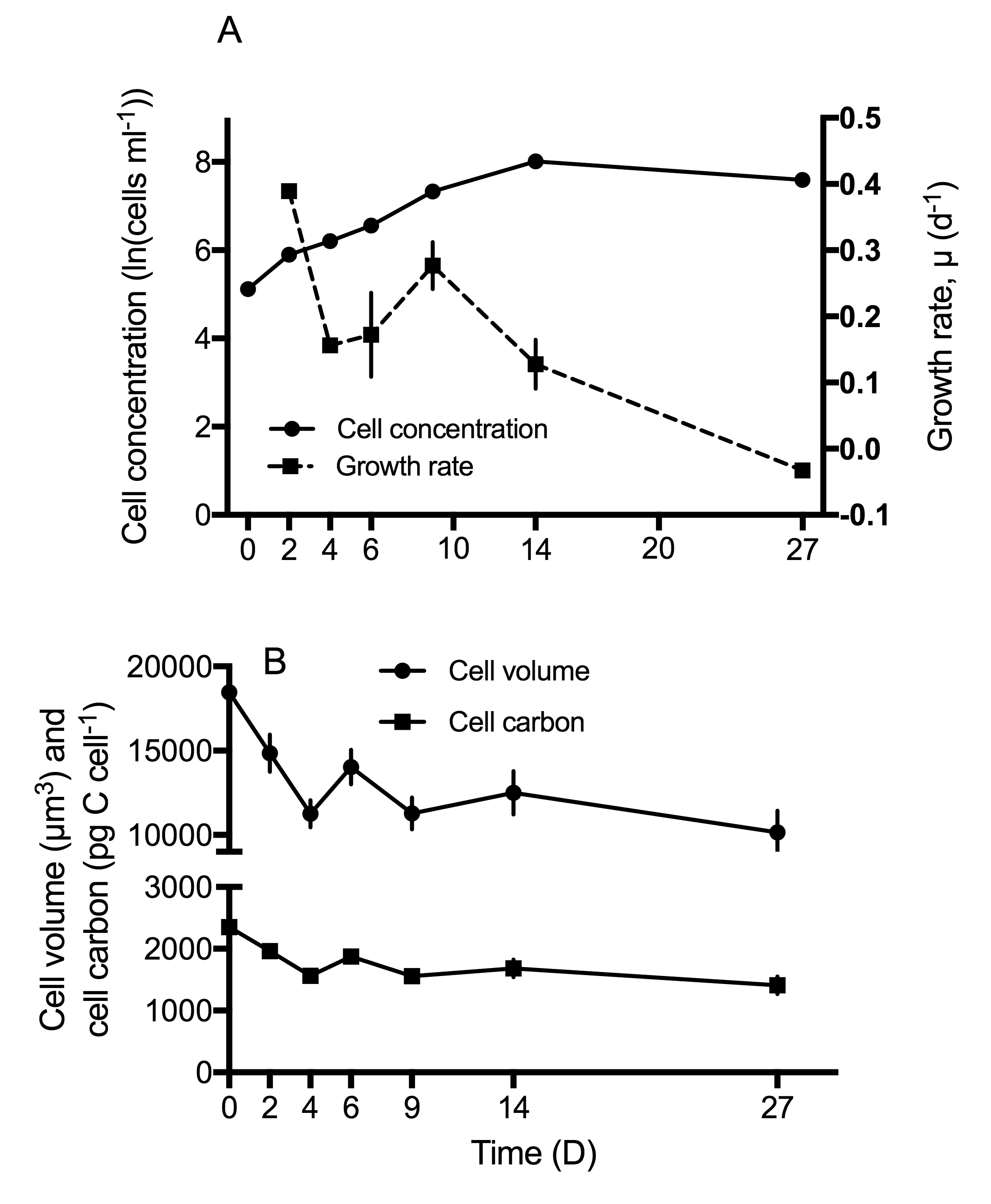

Supplement: S3 Fig — (A) Development in cell concentration and growth rate of N. aeruginosum during prey deprivation. Data points represent means ± SE (n = 8, except day 6 and 9 = where n = 4 and 5 respectively). (B) Cell volume and cell carbon of N. aeruginosum as a function of time under prey deprivation. Data points represent means ± SE (n = 30). (TIFF) [file pone.0181751.s003.tiff]
